# Complete mitochondrial genome of the taxonomically notorious sea star, *Henricia leviuscula* (Asteroidea, Spinulosida, Echinasteridae), from South Korea

**DOI:** 10.1080/23802359.2019.1636731

**Published:** 2019-07-22

**Authors:** Taekjun Lee, Sook Shin

**Affiliations:** a Marine Biological Resource Institute, Sahmyook University, Seoul, Korea;; b Division of Life Sciences, College of Life Sciences and Biotechnology, Korea University, Seoul, Korea;; c Department of Chemistry Life Science, Sahmyook University, Seoul, Korea

**Keywords:** Echinodermata, Asteroidea, phylogeny, mitogenome

## Abstract

In this study, next-generation sequencing was used to obtain the complete mitogenome of *Henricia leviuscula* (Stimpson, 1857). The mitogenome form was found to be a circular molecule 16,119-bp long with a 60.4% AT bias. The gene arrangement of *H. leviuscula* was exactly the same as that of previously reported mitogenome for another species of Echinasteridae, such as *Echinaster* (*Othilia*) *brasiliensis*, containing 37 genes (13 PCGs, 22 tRNAs, and 2 rRNAs). A phylogenetic tree was constructed using the 13 PCGs and 2 rRNA sequences. *Henricia leviuscula* formed a monophyletic clade with *E.* (*O.*) *brasiliensis* and this clade formed a larger clade with species of the Paxillosida and Valvatida.

The genus *Henricia* belongs to the family Echinasteridae and this family is the only taxa at the family level in the order Spinulosida. *Henricia* is broadly distributed in the northern Pacific and has a high species diversity. Therefore, many taxonomists have found the identification and classification of *Henricia* confusing (Verrill 1909; Fisher 1911, 1930; Hayashi 1940; Djakonov 1961; Jewett et al. 2015). Moreover, a number of studies have attempted a detailed identification of *Henricia* based on DNA taxonomy with mitochondrial cytochrome oxidase subunit I (Laakmann et al. 2016; Layton et al. 2016; Knott et al. 2018). However, in these studies, the specific species name could not be assigned. In this study, we carefully identified samples of *Henricia* based on morphological comparison with previous clearly elucidated morphological studies (Hayashi 1940; Djakonov 1950; Clark and Jewett 2010). In addition, we characterized the complete mitochondrial genome sequence of *H. leviuscula* and performed a phylogenetic analysis within the Asteroidea, with data from GenBank.

For this study, a specimen was collected from water adjacent to Pohang, South Korea (36°11′58″N, 129°24′47″E) by scuba diving, at a depth of 18 meters. Voucher specimens and mitochondrial DNA samples were deposited in the Marine Echinoderm Resources Bank of Korea (Seoul, Korea) and granted a voucher number: MERBK-A-1290. Mitochondrial DNA analyses conformed to the method described by Lee and Shin (2018). The phylogenetic analysis was performed with 11 complete mitogenomes of asteroids, including *H. leviuscula*, and two crinoids, *Florometra serratissima* (NC_001878) and *Phanogenia gracilis* (NC_020771), were used as outgroups for this analysis. The 13 PCGs and two rRNAs of mitogenome sequences were using maximum likelihood (ML) with RAxML 8.2 (Stamatakis 2014).

The mitogenome sequence of *H. leviuscula* was 16,119-bp long and was submitted to GenBank under the accession number MK947912. The gene arrangement of *H. leviuscula* was exactly the same as that of previously reported mitogenome for another species of Echinasteridae, such as *Echinaster* (*Othilia*) *brasiliensis* (Seixas et al. 2019). The overall nucleotide composition of *H. leviuscula* was 36.0% A, 25.9% C, 13.7% G, and 24.4% T with a 60.4% AT bias. The nine PCGs were initiated with the start codon (methionine), but ND3 and ND4L initiated with the isoleucine, ATT, and ATC, respectively. Moreover, ND1 and ND2 initiated with CAC, which has been coded to methionine or valine in echinoderms. The 12 PCGs terminated with a termination codon (TAA or TAG); however, CytB had no terminated codon but had phenylalanine (TTC)+T at the 3' end.

To reveal the phylogenetic relationship of *H. leviuscula* within asteroids, a phylogenetic tree was constructed based on the concatenated sequences of 13 PCGs and two rRNA identified using the ML method. The ML tree showed two large clades; *Henricia leviuscula* formed a monophyletic clade with *E.* (*O.*) *brasiliensis*, and this clade formed a larger clade with species of the Paxillosida and Valvatida (Figure 1). Thus, the newly obtained mitogenome in this study expanded the genomic resources available for further evolutionary studies and can play a significant role in conservation genetics.

**Figure 1.**
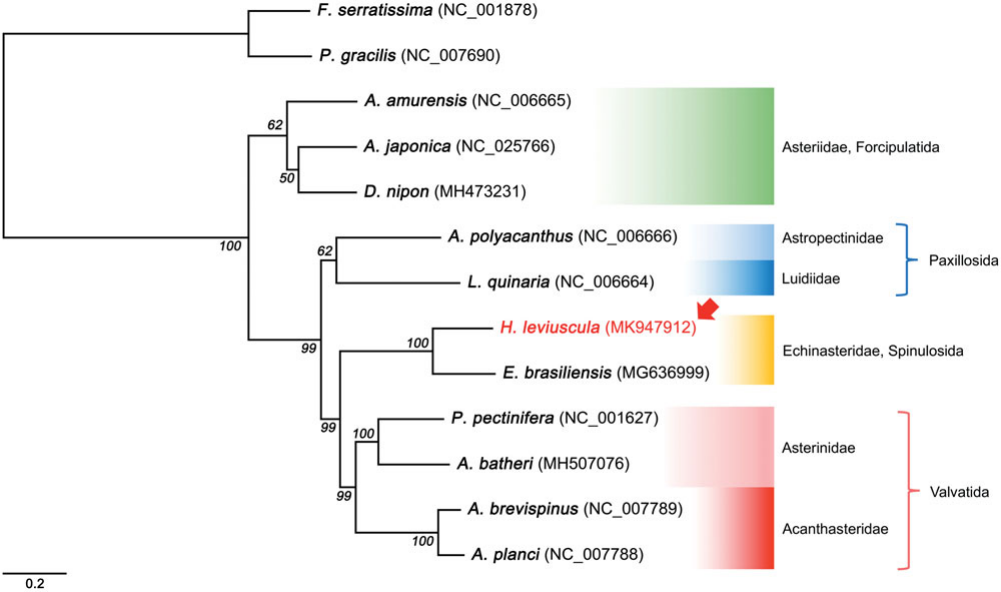
Phylogenetic tree of maximum likelihood (ML) method based on the nucleotide sequences of 13 PCGs and 2 rRNAs of 10 asteroids, included *H. leviuscula* (MK947912), and two crinoids. Bootstrap support values are indicated on each node as >50.
